# Erectile and Clitoral Dysfunction as Harbingers of Cardiovascular Disease: A Perspective

**DOI:** 10.3390/medicina62020416

**Published:** 2026-02-22

**Authors:** Fernanda Priviero, Fiona Hollis, Susan K. Wood, Mark Uline, Karl-Erik Andersson, R. Clinton Webb

**Affiliations:** 1 Cardiovascular Translational Research Center, University of South Carolina, Columbia, SC 29208, USA; fernanda.priviero@uscmed.sc.edu; 2 Department of Cell Biology and Anatomy, University of South Carolina, Columbia, SC 29208, USA; 3 Department of Biomedical Engineering, University of South Carolina, Columbia, SC 29208, USA; 4 Department of Pharmacology, Physiology and Neuroscience, University of South Carolina, Columbia, SC 29208, USA; 5 William Jennings Bryan (WJB) Dorn Veterans Administration Medical Center, Columbia, SC 29208, USA; 6 Division of Clinical Chemistry and Pharmacology, Lund University, 221 00 Lund, Sweden

**Keywords:** sexual dysfunction, erectile dysfunction, female sexual arousal disorder, penis, clitoris, female sexual dysfunction

## Abstract

Sexual dysfunction (SD), more specifically vasculogenic erectile dysfunction (ED) in men and female sexual arousal disorder (FSAD) in women, is increasingly recognized as a marker of cardiovascular disease (CVD). While extensive literature documents vasculogenic ED as an early warning sign of coronary artery disease (CAD) and other atherosclerotic manifestations, the evidence for analogous phenomena in women is emerging but less mature. This perspective explores epidemiologic associations, shared pathophysiologic mechanisms, clinical implications, and screening paradigms for ED and FSAD as cardiovascular (CV) risk-enhancing conditions. This perspective endorses that clinicians should incorporate genital vasculogenic SD into CV risk stratification and that multidisciplinary care (primary care, cardiology, urology/gynecology) is warranted. A summary table outlines key features and actionable steps.

## 1. Introduction

Sexual function is a complex interplay of neurologic, hormonal, vascular, and psychological systems [1]. Dysfunction of these systems may not only impact quality of life but may also reflect systemic vascular pathology. According to the Princeton IV Consensus Recommendations for the Management of Erectile Dysfunction and Cardiovascular Disease, vasculogenic erectile dysfunction (ED) is an independent marker for cardiovascular (CV) risk [2]. In men, the association between vasculogenic ED and cardiovascular disease (CVD) is now well established, with vasculogenic ED regarded as a potential early manifestation of atherosclerosis [3,4,5]. In women, female sexual arousal disorder (FSAD)/female sexual dysfunction (FSD) are less well studied in this context, but recent studies suggest a meaningful association between FSAD and CVD and the importance of addressing FSD in women with CVD [6,7].

Given the global burden of CVD and the often-silent nature of early vascular disease, organic SD presents an under-recognized window of opportunity. In this perspective, we review current evidence, mechanistic rationales, and clinical implications, and propose a framework for integrating SD into CV risk assessment. For this, we performed a non-systematic literature search in PubMed using combinations of the following terms: sexual dysfunction, erectile dysfunction, female sexual dysfunction, female sexual arousal disorder, cardiovascular disease, atherosclerosis, endothelial dysfunction, inflammation, oxidative stress, autonomic dysfunction, menopause, and cardiometabolic risk factors. We prioritized clinically relevant human studies, major consensus/guideline statements, and seminal mechanistic reports, while preclinical evidence was included when needed to corroborate key concepts. This perspective synthesizes the available literature and incorporates the authors’ expert interpretation to propose a conceptual framework, acknowledging that some elements represent informed opinion where definitive evidence is limited.

## 2. Epidemiologic Evidence

### 2.1. ED in Men

Multiple studies and meta-analyses have demonstrated that vasculogenic ED is associated with an increased risk of CVD events. In fact, in 2024, the Princeton IV Consensus Recommendations for the Management of Erectile Dysfunction and Cardiovascular Disease further emphasized that ED should be considered a “risk-enhancing factor” in CV risk stratification [2]. This view is supported by evidence that vasculogenic ED typically precedes coronary artery disease (CAD) by approximately 2–5 years, providing a potential window for earlier intervention [8,9]. The link between these conditions is largely explained by their shared pathophysiological mechanisms, including endothelial dysfunction, oxidative stress, and inflammation. Moreover, accumulating evidence indicates that ED correlates positively with both the incidence and severity of CVD [10,11].

Importantly, the largest prospective and case–control studies that form the basis for current recommendations predominantly evaluated vasculogenic ED, often defined with validated questionnaires and by excluding men with primary psychogenic etiologies. Therefore, the strongest epidemiologic data apply to men with organic/vascular ED, and the strength and nature of the association with CVD may differ in populations where psychogenic or mixed ED is more prevalent.

### 2.2. Female Sexual Arousal Disorder (FSAD) and Female Sexual Dysfunction (FSD)

FSD is defined as a group of disorders of sexual desire, arousal, orgasm, and/or sexual pain that occur during the sexual response cycle and cause significant personal distress or interpersonal difficulty. Within FSD, the term female sexual arousal disorder (FSAD) is used to refer to the genital/clitoral arousal (or engorgement) subtype described in international classifications, characterized by preserved subjective excitement but diminished genital vasocongestion, lubrication, and genital sensations during sexual activity, with associated distress [12]. It is important to highlight this is different from persistent genital arousal disorder/genito-pelvic dysesthesia (PGAD/GPD), in which patients report persistent, unwanted genital arousal sensations occurring independently of sexual desire [13,14].

FSD is a more complex condition and often an underestimated problem. Although in women data are more limited, a recent meta-analysis compiling 54 studies found that subjects with established CVD had a 1.51-fold increased odds of FSD (OR 1.51, 95% CI 1.34–1.69) [7]. Indeed, a study reported that 60% of women with coronary artery disease were diagnosed with FSD [15], reinforcing the correlation of CVD and FSD. While specific clitoral vascular dysfunction studies are sparse, a study found associations between clitoral pulsatility index and metabolic syndrome in women with SD [16]. The increased pulsatility index reflects increased resistance to blood flow associated with microvascular lesions (micro atherosclerosis) [17]. Thus, genital arousal/engorgement dysfunction in women may serve as a proxy for underlying vascular disease, though further longitudinal data are needed.

However, definitions of FSD and the tools used to assess it vary substantially across studies. Many investigations rely on the Female Sexual Function Index (FSFI), while others use alternative instruments or non-validated questionnaires, often with different cut-offs for “dysfunction” and different weighting of desire, arousal, lubrication, orgasm, pain, and satisfaction domains. In addition, mood disorders, menopausal status, relationship factors, and concomitant medications are frequently under-reported or incompletely adjusted for, which may influence the magnitude and even the direction of the observed associations between FSD and CVD.

### 2.3. Methodological Considerations and Limitations of Epidemiologic Data

While the overall body of evidence supports an association between SD and CVD, several methodological issues should be considered when interpreting these data. First, there is substantial heterogeneity in how SD is defined, and the complexity of its assessment is reflected in the large number of accepted, validated instruments currently in use [18]. In men, some studies use multi-item instruments (e.g., IIEF-derived scores) with established cut-offs, whereas others rely on single questions or non-validated scales. In women, the use of FSFI versus other questionnaires, as well as variable thresholds for defining FSD, leads to differences in sensitivity and specificity. This variability can inflate heterogeneity across studies and makes direct comparison of effect sizes challenging.

Second, most large epidemiologic cohorts summarized in our review focus on vasculogenic or organic ED, often excluding men with predominantly psychogenic ED. This improves mechanistic plausibility regarding a shared vascular substrate with CVD, but it also narrows generalizability. Patients whose SD is primarily driven by psychological, relational, or iatrogenic factors may not exhibit the same degree of CV risk as those with vasculogenic ED.

Third, CV populations have a high burden of depression, anxiety, polypharmacy, and multimorbidity. Antihypertensives, antidepressants, antipsychotics, and other medications can contribute to SD [19], as can other comorbidities such as heart failure symptoms, diabetes, obesity, and chronic kidney disease. Although many studies adjust for some of these factors, residual confounding cannot be ruled out. Additionally, depression can both worsen sexual function [20] and independently increase CV risk [21,22], and hence, a poor assessment of these conditions may exaggerate the apparent link between SD and CVD. Conversely, when sexual side effects of medications are not distinguished from vascular dysfunction, as well as when depression is induced by SD [23], the true strength of the association between vasculogenic SD and CVD may be underestimated.

These limitations suggest that the observed relationships between SD and CVD risk may be inflated in some settings and obscured in others, depending on how SD is defined, which tools are used, and how carefully comorbidities, mood disorders, and medications are accounted for. Recognizing these nuances is critical to properly differentiate the conditions that might be associated with CVD risk.

Taking these limitations into consideration, the epidemiologic data and the mechanistic evidence summarized in Table 1 support SD, particularly vasculogenic ED, as a potential early clinical signal of systemic vascular disease.

## 3. Pathophysiological Mechanisms Connecting ED and CVD

The vascular system underpinning genital arousal in both sexes shares essential features with the CV system. Key mechanistic links include endothelial dysfunction, impaired arterial flow in the small pudendal arteries due to atheroma plaques, metabolic and cellular disturbances, excessive sympathetic activity, and other shared risk factors encompassed within the CV–kidney–metabolic syndrome. While endothelial dysfunction is a well-recognized contributor to both ED and CVD, genital organs are specialized neurovascular tissues that undergo a rapid state change from a high-resistance (flaccid or non-aroused) condition to a low-resistance (aroused) condition. In the penis and clitoris, arousal depends on coordinated dilation of inflow arteries (internal pudendal/cavernosal and helicine arteries) together with relaxation of trabecular/cavernosal smooth muscle, permitting sinusoidal filling and tissue expansion. In the penis, full rigidity additionally requires an effective veno-occlusive mechanism provided by compression of subtunical venules against the tunica albuginea to restrict venous outflow. Thus, SD may result from reduced arterial inflow, impaired neurogenic vasorelaxation, altered smooth-muscle tone, and/or structural remodeling (fibrosis) that disrupts sinusoidal compliance and (in men) veno-occlusion. However, the convergent downstream pathway across several of these processes is reduced nitric oxide (NO) bioavailability, which plays a pivotal role in mediating genital engorgement [42,43]. Released from endothelial cells or nitrergic neurons, NO induces vasodilation in penile (and, by extension, clitoral) arteries. Accordingly, reduced NO bioavailability reflects systemic endothelial dysfunction, a common underlying mechanism linking ED and CVD [8,44]. Moreover, NO bioavailability and endothelial function are further impaired by factors such as inflammation and oxidative stress, both known to exacerbate endothelial dysfunction and diminish NO signaling in these conditions [10,45,46].

Physiologically, penile and clitoral erectile tissues are maintained in the flaccid/non-aroused state by tonic sympathetic (adrenergic) activity, in which norepinephrine-dependent contraction of cavernosal and vascular smooth muscle preserves high resistance/low inflow, while sexual arousal requires dominance of parasympathetic relaxant mechanisms over this sympathetically driven tone, mechanisms that are supported in both clinical [47,48,49,50] and preclinical studies. Therefore, increased sympathetic activity is another critical driver of SD that often coexists with CV risk factors, including hypertension and stress. In women, decreased heart rate variability (HRV, which is a marker of reduced parasympathetic and increased sympathetic tone) has been associated with increased reports of FSAD and overall SD [51]. A low HRV implies a shift to a dominant sympathetic tone [14], and hence, a higher sympathetic tone will favor the detumescent state of the sexual organs. Similarly, in men, higher sympathetic tone and lower parasympathetic activity has been reported in ED patients [52].

In women, another key factor that deserves attention is that menopause is a period marked by increased CV risk [53] and a heightened susceptibility to FSD [31,54,55], both being often attributed to the lower levels of estrogen. Mechanistically, the decline in ovarian hormones during the menopause transition may contribute to autonomic imbalance, with multiple studies showing reduced cardiac vagal modulation/HRV and a shift toward relatively greater sympathetic influence after menopause [56]. Consistent with this, vasomotor symptoms are accompanied by acute reductions in HF-HRV (cardiac vagal control), suggesting transient parasympathetic withdrawal as part of the physiology linking menopause symptoms to cardiometabolic and vascular risk [57,58,59]. In parallel, estrogen deficiency drives vulvovaginal tissue changes and altered genital sensation that contribute to FSD, particularly pain and arousal/lubrication difficulties [60].

Other CV risk factors such as dyslipidemia, diabetes mellitus, obesity, smoking, metabolic syndrome, and sedentary lifestyle are widely recognized contributors to SD [3,7,8,61]. In atherosclerosis, the small caliber of the vessels supplying blood to the genital organs makes them particularly susceptible to atheroma formation, potentially leading to early manifestations of SD before cardiac symptoms appear [4,11,30]. The arteries supplying the corpora cavernosa have a smaller lumen diameter (1–2 mm) compared to coronary arteries [4]. In women, a study demonstrated that clitoral arteries are even smaller in diameter, ranging from 0.7 to 1.1 mm, which may predispose them to flow limitation at an earlier stage [62]. Additionally, in this population, further complexity exists due to hormonal variation (menopause, estrogen deficiency), pelvic vascular anatomy, and co-morbidities such as pelvic floor dysfunction, which likely modulate the link between FSD and CVD [63]. Table 1 summarizes the findings from several studies associating SD and CVD in men and women.

## 4. Clinical Implications: Screening, Risk Stratification, and Management

### 4.1. Screening for CV Risk in Patients with SD

As summarized in Table 1, SD (especially vasculogenic ED) clusters with established cardiometabolic risk factors and vascular dysfunction, supporting its consideration in CV risk discussions in appropriate patients. Therefore, there has been a growing call among clinicians to use SD as a clinical window for CV risk screening. In men, Brock (2014) noted that SD may often be the only reason a patient seeks medical attention, providing an important opportunity to assess CV risk [5]. The 2024 Princeton IV Consensus further recommends that clinicians should regard all patients with SD as being at risk for cardiac events until proven otherwise [2]. Consistently, the 5th International Consultation on Sexual Medicine endorsed the Princeton Consensus, strongly recommending referral for cardiac evaluation in men with documented vasculogenic ED or those considered at high CV risk [64].

On the other hand, no equivalent formal recommendation or guideline for women with FSD has yet been endorsed by any major consensus or society, despite clear evidence linking clitoral or genital arousal dysfunction, or FSD more broadly, to increased CV risk, which should prompt consideration of CV risk evaluation. Given that FSD is often underdiagnosed and multifactorial, a bidirectional approach should be considered: the diagnosis of FSD should prompt evaluation for CVD risk factors, and conversely, women with CVD should be assessed for FSD and related vascular dysfunction [7].

Nevertheless, it is important to highlight that, in both sexes, SD is frequently multifactorial (hormonal, neurologic, psychogenic/relational, medication-related), and therefore, CV evaluation should be targeted and interpreted in clinical context rather than applied as universal screening.

### 4.2. Clinical Management Considerations

Lifestyle modifications remain the most effective intervention targeting modifiable risk factors, such as physical activity, healthy diet, smoking cessation, and weight loss, providing benefits for both CV health and sexual function. A randomized meta-analysis demonstrated that lifestyle changes significantly improved ED while reducing overall CV risk [65]. Consistently, 6-month lifestyle interventions have shown to improve sexual function in both men [66] and women [67].

Regarding pharmacological approaches, for men, phosphodiesterase type 5 (PDE5) inhibitors remain the first line therapy for ED after lifestyle recommendations. Importantly, in stable CVD patients not on nitrates, they are generally safe and may even confer vascular benefit [2,64]. In contrast, because FSD is a multifactorial dysfunction that affects desire, arousal, and orgasm, there is no current first line therapy recommended to treat this condition in women. Although PDE5 inhibitors are considered safe for women and have been tested for several other conditions (pulmonary hypertension, infertility, preeclampsia, etc.) [68], a clinical trial showed that sildenafil (the first FDA approved PDE5 inhibitor) failed to improve physical response during sexual activity in women with sexual arousal disorder [69]. Therefore, clinical guidance emphasizes foundational interventions such as education, lifestyle modification, sexual/psychosexual counselling, and partner/relationship assessment to determine underlying causes (such as hormonal, vascular, neurologic) before pharmacological or hormonal therapies (flibanserin, testosterone) should be considered, and hence, tailored to the specific subtype of FSD and patient preference [60].

The clinical management of SD should adopt a multidisciplinary approach, involving urologists/andrologists, gynecologists, cardiologists, and primary care providers in a coordinated effort to assess and address CV risk, aiming not only to relieve sexual symptoms but also to mitigate underlying vascular risk. In men, sexual (dys)function is more frequently discussed and, as mentioned above, is often the primary reason men seek medical attention [5]. In contrast, women’s sexual health is less consistently addressed in clinical settings. An early study highlighted that obstetricians and gynecologists tend to narrow their approach to sexual health to matters of sexual activity, while topics such as sexual identity, orientation, satisfaction, pleasure, and dysfunction are often excluded from the discussion [70]. This raises concern, as the failure to identify SD eliminates the opportunity for referral to CV assessment, thereby missing a potential window for early intervention. Therefore, more recent recommendations emphasize that clinicians and researchers should adopt a broader framework, one that actively promotes sexual wellness and supports fulfilling sexual lives for all individuals, regardless of gender or sex [71]. It is important to note that patients often do not volunteer information about sexual symptoms unless specifically asked [72]; therefore, clinicians, including primary care providers, should take an active role in addressing sexual health, incorporating it systematically into the evaluation of vascular risk [2,68,72]. It is also important to emphasize that inflammation, whether acute, subacute, or chronic, is increasingly recognized as a root cause of SD. Hence, because inflammation is a central pathogenic driver shared by both sexual disorders and other non-communicable diseases (NCDs), sexual health should be addressed in patients with known inflammatory NCDs [73]. It is important to highlight that even in otherwise healthy men and women, systemic inflammation has been associated with organic SD, independent of other comorbidities [11,74,75]. Additionally, in men, inflammation levels positively correlate with ED severity, and comorbidities that increase systemic inflammation (such as obesity and hypertension) are also associated with more severe ED [11,76]. Although evidence in women remains limited, this area is gaining increasing attention, and new findings are beginning to emerge. Interestingly, a recent study demonstrated that in women, inflammation is correlated with FSD in patients with depression, shedding light on mechanistic pathways that may help drive organic FSD even when the dysfunction is considered primarily psychogenic [77]. These findings suggest shared inflammatory mechanisms contributing to FSD across etiologies, whether of psychogenic or vasculogenic origin. Corroborating this concept of inflammation and infection, evidence also suggests that men and women diagnosed with periodontitis should be evaluated for SD, and vice versa [78,79,80,81]. Notably, this bidirectional hypothesis gained traction after epidemiologic and scientific-statement evidence linked periodontitis with atherosclerotic CVD risk [82]. Therefore, according to the clinical algorithm proposed by the Princeton IV Consensus, patients presenting with ED should undergo a detailed sexual history (onset, progression, comorbidities, and medications) and evaluation of CV risk factors, including blood pressure, lipid profile, glucose, smoking status, and family history. Although women-specific guidance is less definitive than for men, chronic inflammatory disorders such as systemic lupus erythematosus and rheumatoid arthritis are well established to confer higher CV risk [83,84]. Accordingly, FSD in women, when accompanied by systemic inflammation, should be considered a potential CV risk marker and should prompt formal CV risk assessment. If CVD risk is high or symptoms suggest CAD or other vascular disease, patients should be referred to a cardiologist for further assessment of the CV health condition. Lifestyle interventions should be initiated, and comorbidities optimally managed. For men, PDE5 inhibitors should be considered when appropriate. Sexual and CV outcomes should be monitored and risk-factor management intensified if SD persists or worsens despite treatment [2,68].

## 5. Discussion

**Figure 1:**
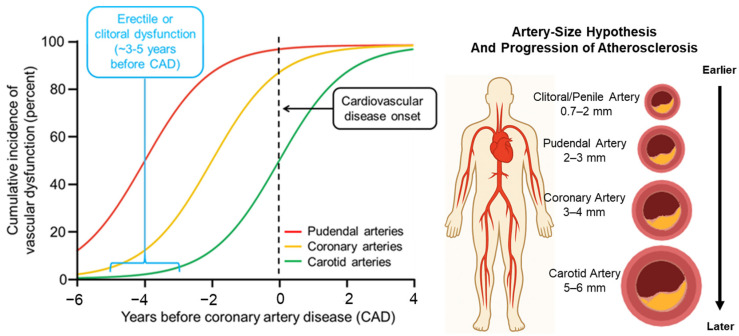
The artery size hypothesis [4]: Sexual dysfunction (SD) as an early vascular signal. Systemic endothelial dysfunction/atherosclerosis may produce earlier flow-limiting effects in smaller genital arteries (~0.7–2 mm) than in larger coronary (~3–4 mm) or carotid (~5–6 mm) arteries. SD can therefore precede overt coronary symptoms by ~3–5 years, highlighting an opportunity to consider earlier cardiovascular risk assessment. Created with Biorender.com and ChatGPT 5.1 (OpenAI, San Francisco, CA, USA).

The conceptual shift toward recognizing SD (particularly ED and clitoral/genital arousal dysfunction) as a sentinel marker of vascular health has emerged gradually over time. To illustrate this evolution of the field, we retained select seminal studies from earlier decades for historical context and, where available, we included contemporary cohorts, meta-analyses, and guidelines that corroborate these foundational observations. Notably, comparable longitudinal evidence in women remains limited. In this context, in men, although isolated studies had suggested this connection earlier [4,5], guidelines and consensus statements have just begun to emphasize it more explicitly and systematically [2,61]. On the other hand, in women, the complexity of this condition has slowed progress in understanding the causal relationship between FSD and CVD, and therefore, more in-depth investigation is needed to better establish causality. Figure 1 illustrates how arterial caliber affects the development of SD and its temporal relationship to overt CVD. For many years, particularly in men, ED was regarded primarily as a consequence of CV disease rather than an early manifestation of it, overlooking subtle indications that it often precedes and predicts subsequent CV complications. The nuanced nature of FSD has made this inference even more challenging in women, with research now beginning to mirror the male trend in exploring the association between FSD and CV risk [7].

In men, the evidence is robust. ED portends CAD, stroke, and CV mortality. The time-lag of several years between ED onset and clinical CAD provides clinicians an actionable interval. That is, a middle-aged man who reports new onset ED and has otherwise moderate CV risk factors may represent a “latent CVD” patient who merits more intensive investigation and risk-factor modification [40,41].

In women, the picture is less mature but evolving. The meta-analysis demonstrating a ~1.5-fold increased odds of FSD in women with CVD is compelling [7]. The vascular underpinning of genital arousal (including clitoral engorgement) means that vascular insufficiency [85] may manifest as SD prior to overt CVD [7]. Given the historical under-assessment of FSD and pelvic vascular health in CV research, this remains fertile ground. It suggests that gynecologists, urologists, and cardiologists should collaborate to address SD as a potential CV harbinger in women. Special attention should be given to the changes occurring during the perimenopausal period, as this may represent an important window for strategic interventions [86], as well to women with other immune/inflammatory conditions [77].

Pragmatically, integrating inquiry about sexual function into CV risk assessment may open the door to earlier identification of high-risk individuals. For example, taking a sexual history, including onset, severity, progression, and associated vascular risk factors, could be incorporated into routine visits in middle-aged adults. Likewise, in clinical trials of new pharmacological agents, where CV monitoring is routinely required, assessment of sexual function should also be incorporated, as it may provide an additional and sensitive window into both adverse and beneficial vascular effects of the intervention.

Nevertheless, several caveats apply. SD can have non-vascular causes (neurologic, hormonal, psychogenic), particularly in women, and the specificity for underlying vascular disease is limited. Moreover, there is no consensus yet on how strongly SD should influence CV screening protocols. Additionally, in women, there is heterogeneity of SD phenotypes that complicate translational application [87]. Longitudinal data linking female genital vascular dysfunction to hard CV endpoints are scarce.

From a research perspective, future directions include:

- Large-scale prospective studies in men and women assessing SD (vascular subtype) and incident CVD outcomes stratified by age, sex, ethnicity, and comorbidities.
- Refinement of vascular markers of genital arousal dysfunction (such as Doppler indices of clitoral or penile vessels) and their predictive value for CVD.
- Evaluation of sexual function during clinical trials of drugs in general (not only related to CVD or SD).
- Development of sex-specific guidelines on how SD should inform CV work-up and management.

## 6. Conclusions

SD (manifesting as vasculogenic ED in men and FSAD in women) should no longer be regarded merely as a quality-of-life concern. In men, the evidence supports vasculogenic ED as a clinical marker that can precede overt CVD and should prompt cardiometabolic risk evaluation and management of modifiable risk factors. In women, FSAD is compellingly associated with CV risk, but the evidence base is more limited and heterogeneous, and longitudinal data linking objective genital vascular measures to incident CVD outcomes remain scarce. Therefore, FSAD should currently be viewed as a risk-associated clinical signal rather than a proven predictive marker. Clinicians across disciplines, including urology, gynecology, cardiology, and primary care, should collaborate to incorporate sexual history into CV risk stratification, recognizing sex-specific mechanisms and contributors (menopause, autonomic imbalance, psychosocial factors, pelvic comorbidities) and initiating early management of modifiable risk factors. Likewise, oral health professionals and physicians should recognize the emerging association between periodontitis and organic SD. Ultimately, as the evidence base expands, prospective studies integrating validated sexual function measures with objective genital vascular metrics and hard CV outcomes will be essential to define how SD (particularly in women) can be incorporated into CVD prevention strategies.

## Figures and Tables

**Table 1:** Sexual dysfunction (SD) has been consistently linked to cardiovascular diseases (CVD) and may manifest before the clinical presentation of overt CV symptoms.

Authors and Year	Population	Summary
Wabrek et al., 1980 [24]	(M)	In 131 men hospitalized for acute myocardial infarction (MI), 66% reported significant pre-MI SD.
Jaarsma et al., 1996 [25]	(M & F)	In 62 patients with advanced heart failure, 37% were unable to perform sexually. SD correlated significantly with symptom severity and reduced exercise tolerance but not with ejection fraction.
Greenstein et al., 1997 [26]	(M)	In 40 men undergoing coronary angiography, ED severity correlated significantly with the number of diseased coronary vessels. Hypertension, diabetes, and age further worsened erectile quality.
Drory et al., 1998 [27]	(M)	In men, sexual activity after MI was reduced in frequency and satisfaction, most strongly predicted by pre-MI sexual frequency and satisfaction; age, diabetes, and depression negatively affected post-MI sexual activity.
Hultgren et al., 1999 [28]	(F)	Women with aortoiliac occlusive disease (AIOD) presented significant impairment of female sexual health, with both vascular insufficiency and surgical factors contributing.
Burchardt et al., 2001 [29]	(M)	In hypertensive men, 70.6% reported ED, associated with MI, heart failure, or stroke.
Montorsi et al., 2003 [30]	(M)	In men with acute chest pain and angiographically documented CAD, 49% had ED, and 67% of those reported ED onset preceding angina or myocardial ischemia symptoms by an average of 39 months.
Addis et al., 2005 [31]	(F)	In postmenopausal women with CHD, 65% of sexually active women reported ≥1 sexual problem. In older women with CVD, SD is prevalent and multifactorial, involving both psychological and CV determinants.
Shi et al., 2006 [32]	(M)	In men, the prevalence of ED increased, and sexual frequency decreased 6 months before coronary stenting. ED was significantly associated with age, diabetes, multivessel coronary disease, and current smoking. Nearly 49% reported ED onset preceding coronary symptoms by a mean of 33 months.
Eyada et al., 2007 [33]	(F)	In women hospitalized for unstable angina or non-ST-elevation MI (NSTEMI), only 48.6% resumed sexual activity within 12 weeks of discharge, and most reported marked dissatisfaction or reduced frequency. SD severity was correlated with depression, anxiety, and lack of cardiac rehabilitation participation.
Kaya et al., 2007 [15]	(F)	In women with CAD and 15 healthy controls, FSD was present in 60% of CAD patients vs. 33% of controls. All FSFI domains except satisfaction (desire, arousal, lubrication, orgasm, pain) were significantly reduced.
Cook et al., 2008 [34]	(M)	In men with congenital heart disease, ED prevalence was 38%, independent of congenital heart disease complexity.
Schwarz et al., 2008 [35]	(M & F)	In patients (76 men, 24 women) with chronic compensated heart failure (NYHA I–III), 84% of men had ED and 87% of women had FSD. In this study, SD was highly prevalent in both sexes with chronic heart failure, often preceding cardiac symptoms.
Hoffman et al., 2010 [36]	(M)	In clinically depressed, sedentary men, ED severity correlated significantly with higher Framingham CV risk scores and lower flow-mediated dilation, supporting that vascular (not psychological) mechanisms underlie ED in depressed men.
Lemogne et al., 2010 [37]	(M)	In men with CHD, 57.6% had significant ED. Depressive mood and hypertension were independent predictors of ED.
Kriston et al., 2010 [38]	(M & F)	In patients undergoing cardiac rehabilitation, moderate-to-severe SD was present in 20–40% of men and 43–50% of women, while moderate-to-severe depressive symptoms occurred in 14–17%.
Vlachopoulos et al., 2013 [39]	(M)	ED affects ≈40% of men over age 40 and shares major risk factors with CVD. ED frequently precedes clinical CAD by 2–5 years and independently increases the risk of future CV events by 44%.
Rinkūnienė et al., 2021 [40]	(M)	In men after MI, the prevalence of ED was 62%. All participants had at least one CV risk factor. ED is common in post-MI patients and closely associated with age and hypertension, reinforcing its role as a vascular marker of CV disease.
Mostafaei et al., 2023 [41]	(M)	ED independently increases CV risk. Men with ED had higher risks of CVD events, CHD, CV mortality, all-cause mortality, MI, and stroke.
Dilixiati et al., 2024 [7]	(F)	CVD increases the risk of FSD by 51%. Among CVD subtypes, hypertension, stroke, and MI showed the strongest associations with FSD.

Table abbreviations: F: female; M: male; MI: myocardial infarction; SD: sexual dysfunction; ED: erectile dysfunction; AIOD: aortoiliac occlusive disease; CV: cardiovascular; CHD: coronary heart disease; CAD: coronary artery disease; FSD: female sexual dysfunction.

## Abbreviations

The following abbreviations are used in this manuscript:

AIOD	Aortoiliac occlusive disease
CAD	Coronary artery disease
CAC	Coronary artery calcium
CVD	Cardiovascular disease
CHD	Coronary heart disease
CV	Cardiovascular
ED	Erectile dysfunction
FSAD	Female sexual arousal disorder
FSD	Female sexual dysfunction
FSFI	Female Sexual Function Index
HRV	Heart rate variability
ICSM	International Consultation on Sexual Medicine
ISSWSH	International Society for the Study of Women’s Sexual Health
LDL	Low-density lipoprotein
MI	Myocardial infarction
NCD	Non-communicable disease
NO	Nitric oxide
NSTEMI	Non–ST-elevation myocardial infarction
OR	Odds ratio
PDE5	Phosphodiesterase type 5
PGAD	Persistent genital arousal disorder
RCT	Randomized controlled trial
SD	Sexual dysfunction
